# Losses in the Royal Army Medical Corps

**Published:** 1914-09-19

**Authors:** 


### ROUND THE WORLD.

[The Editor Invites Early Information and Contributions Marked "Fellow-Workers."]

### Losses in the Royal Army Medical Corps.

As on previous occasions the gallant officers of the Royal Army Medical Corps have paid a sad toll on the field of battle, but it is to be hoped that a good many of those reported missing will eventually be found unharmed. In the course of such a remarkable strategic retreat as has been carried out by the allied forces recently, it was almost inevitable that a number of medical officers should be left behind in pursuit of their duties. One can only hope that the Red Cross, under which they doubtless collected their wounded, sufficiently protected them from the attentions of the enemy. So far it appears that only one R.A.M.C. officer has been killed, although several have been wounded.

The first Army surgeon to fall a victim to the bullets of the enemy was Captain Augustus Scott Williams, an old St. Bartholomew's man, who qualified M.R.C.S., L.R.C.P., in 1905, and subsequently worked at the West London Hospital as house-surgeon. Captain Williams joined the R.A.M.C. a year after qualification, and was promoted to the rank of captain five years ago.

Of the wounded, Captain Malcolm Leckie is an old Guy's man who qualified M.R.C.S., L.R.C.P. in 1907, whilst Captain H. J. Perry qualified from Queen's College, Cork, by taking the diplomas of L.R.C.P. I., L.R.C.S. I., and L.M. in 1906.

Among the list of wounded officers admitted to King Edward the Seventh's Hospital has been Captain W. McConaghy, an old Edinburgh University man who took the degrees of M.B., Ch.B. Ed. just ten years ago. Also Lieut. W. K. Morrison, who qualified M.B., Ch.B. Ed. last year, subsequently to which he held the post of house surgeon at the Royal Infirmary, Edinburgh.

The first naval surgeon to be stricken down has been Staff-Surgeon Thomas Aubrey Smyth, who qualified as a graduate of Edinburgh University in 1901, and almost immediately joined the South African Field Force as civil surgeon. Subsequently entering the Navy, he was appointed to H.M.S. *Pathfinder* about ten months ago, and received serious injuries in the lamentable catastrophe which befell that vessel the week before last.

The medical officers of the military forces reported as missing include Lieut.-Col. H. N. Thompson, a distinguished member of the Army Medical Service, who graduated from Dublin University in 1883 and whose honours include the D.S.O. Also Major F. S. Irvine, who has not been seen since the fighting at Mons, which, as a matter of fact, was by no means the first occasion he was under fire, as he served with the South African Field Force in the Boer War and was present at the siege of Ladysmith as well as in numerous other actions.

